# Cost-effectiveness of using the Cervex-Brush (broom) compared to the elongated spatula for collection of conventional cervical cytology samples within a high-burden HIV setting: a model-based analysis

**DOI:** 10.1186/s12913-015-1163-y

**Published:** 2015-11-06

**Authors:** Kathryn Schnippel, Pamela Michelow, Carla J. Chibwesha, Caroline Makura, Naomi Lince-Deroche, Bridgette Goeieman, Masangu Mulongo, Suzette Jordaan, Cynthia Firnhaber

**Affiliations:** Right to Care, Johannesburg, South Africa; Clinical HIV Research Unit, Department of Clinical Medicine, Faculty of Health Sciences, University of the Witwatersrand, Johannesburg, South Africa; Cytology Unit, Department of Anatomical Pathology, Faculty of Health Sciences, University of the Witwatersrand, Johannesburg, South Africa; National Health Laboratory Service, Johannesburg, South Africa; Department of Obstetrics and Gynecology, School of Medicine, University of North Carolina at Chapel Hill, Chapel Hill, North Carolina USA; Health Economics and Epidemiology Research Office, Department of Clinical Medicine, Faculty of Health Sciences, University of the Witwatersrand, Johannesburg, South Africa

## Abstract

**Background:**

From 2010 to 2014, approximately 2 million Pap smears from HIV-infected women were submitted to the South African National Health Laboratory Services (NHLS) through the national cervical cancer screening programme. The objective of this analysis was to determine whether using the plastic Cervex brush (“broom”) would be a cost-effective approach to improve cytology specimen quality as compared to the wooden spatula used currently.

**Methods:**

A decision analysis model was built using the expected adequacy rates for samples collected with the spatula (<$0.02) and broom ($0.23) and the probability of detecting cervical dysplasia. NHLS data was used for testing volumes and rates of HIV-positivity, suitability of specimens, and presence of endocervical cells. Expected positivity of Pap smears in HIV-infected women (73 %), odds ratios of the effectiveness of the broom (OR: 1.57), and improved sensitivity when endocervical cells present (OR: 1.89) are from literature. NHLS costs were used for the collection devices and conventional cytology ($4.89). Cost of clinic visit is from WHO CHOICE ($8.36).

**Results:**

In 2010, 80 % of specimens submitted to NHLS were adequate for evaluation; in 2014, only 54 % met the same criteria. For HIV-infected women, according to the guidelines model, using the wooden spatula costs $6.25 million per year, $16.79 per woman tested. Under intended practice, for each additional HSIL case detected among HIV-infected women, the South African cervical cancer screening programme could save $13.64 (95 % CI: $13.52 to $13.76) by using the broom as its standard of care collection device through increased collection of endocervical cells and consequent reduction in repeat Pap smears.

**Conclusion:**

Under a wide range of parameters tested using a simulation model, the more expensive plastic broom could save the South African cervical cancer screening programme money and increase detection of high-grade cervical dysplasia in HIV-infected women compared to the current wooden spatula.

## Background

Cervical cancer is the second most common cancer among women in South Africa, with an estimated incidence of 31.7 per 100,000 [1]. In the same year (2012), cervical cancer had the highest mortality rate (18.0 per 100,000 women) of cancers among women in South Africa [1]. Cervical cancer is caused by infection with sexually transmitted human papillomavirus (HPV); there is a growing body of evidence of inter-related risks and burdens of HPV and HIV. An estimated 6.3 million persons in South Africa are HIV-infected, women account for approximately 55 % of prevalent HIV infections [2]. A recent systematic review confirmed women who are HIV-infected have a higher incidence of cervical dysplasia, develop cervical dysplasia at an earlier age, are at higher risk of persistent HPV infection, are likely to develop cervical dysplasia earlier, and are at a higher risk of progression of cervical lesions [3]. The conventional Papanicolaou test, or Pap smear, is the standard of care in the public sector in South Africa to screen for cervical cancer [4].

**Fig. 1:**
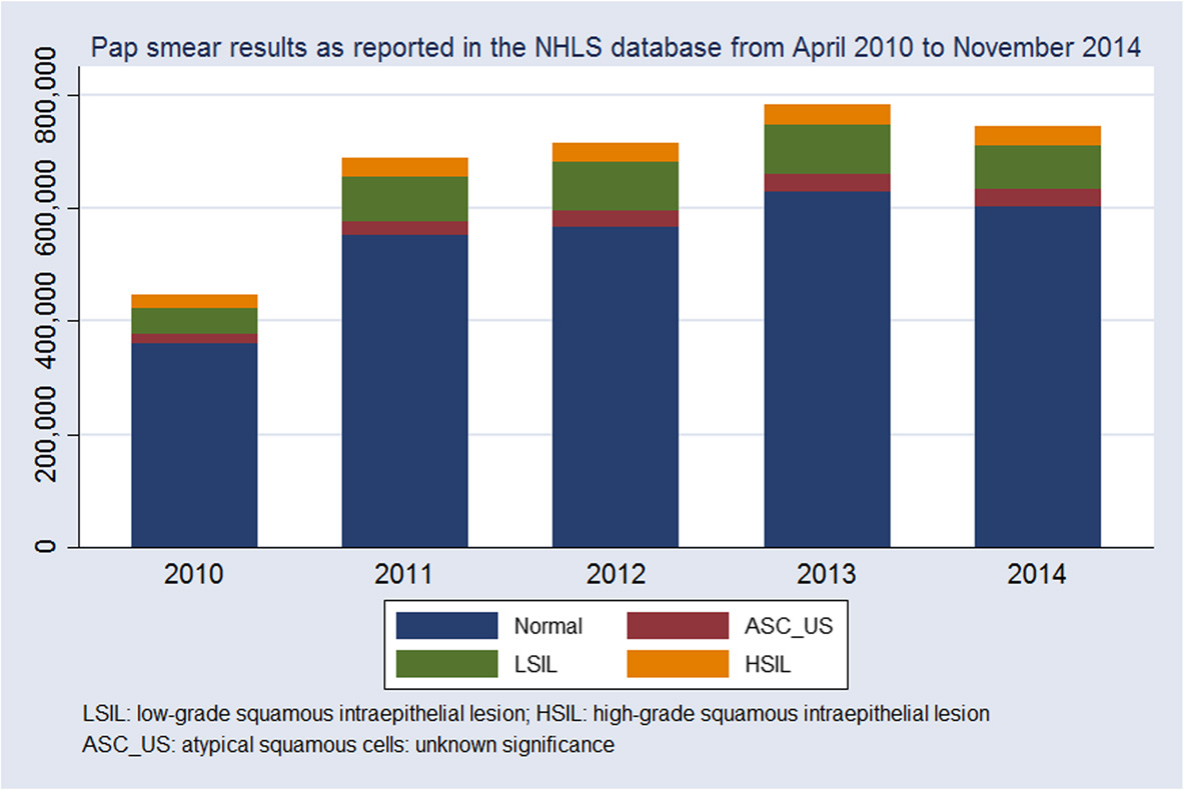
Counts of normal and abnormal Pap smears reported in the South African public sector, April 2010 to November 2014

Recognizing the higher risks of cervical cancer faced by HIV-infected women, a dual programme for cervical screening exists in South Africa. Women who are HIV-negative should be screened 3 times in their lifetime, once every 10 years, from age 30 [4]. From April 2010, the South African guidelines for the management of adults and adolescents with HIV indicate that from age 18 years, women who are HIV-infected should have a Pap smear for cervical cancer screening at HIV diagnosis and, if normal, a repeat Pap smear once every three years [5]. The National Health Laboratory Services (NHLS) has maintained a database of all Pap smears since the start of the HIV screening programme. By the end of 2010, the NHLS had reviewed 600,688 slides of cytological specimens collected from across the 4,000 public health clinics and centres in South Africa. More than 3.6 million slides had been reviewed by the end of November 2014; an estimated 45 to 55 % of which were from HIV-infected women. Figure 1 shows the increase in Pap smears in the South African public sector from 2010 to 2014 as well as the proportion of abnormal smears detected by year.

The South African cervical cancer screening guidelines use the Bethesda guidelines to define smear adequacy. In this system, a slide is considered adequate if it has proper patient identification, is not irreparably broken, has 8,000–12,000 well-preserved squamous cells, has at least 10 well-preserved endocervical or metaplastic cells and less than 75 % of all cells are obscured by blood or inflammation [6]. In conventional cytology, the presence of endocervical or metaplastic cells is one indication that the transformation zone has been sampled and therefore that the sample collected is adequate for the detection of the precursors of cervical cancer [7].

**Fig. 2:**
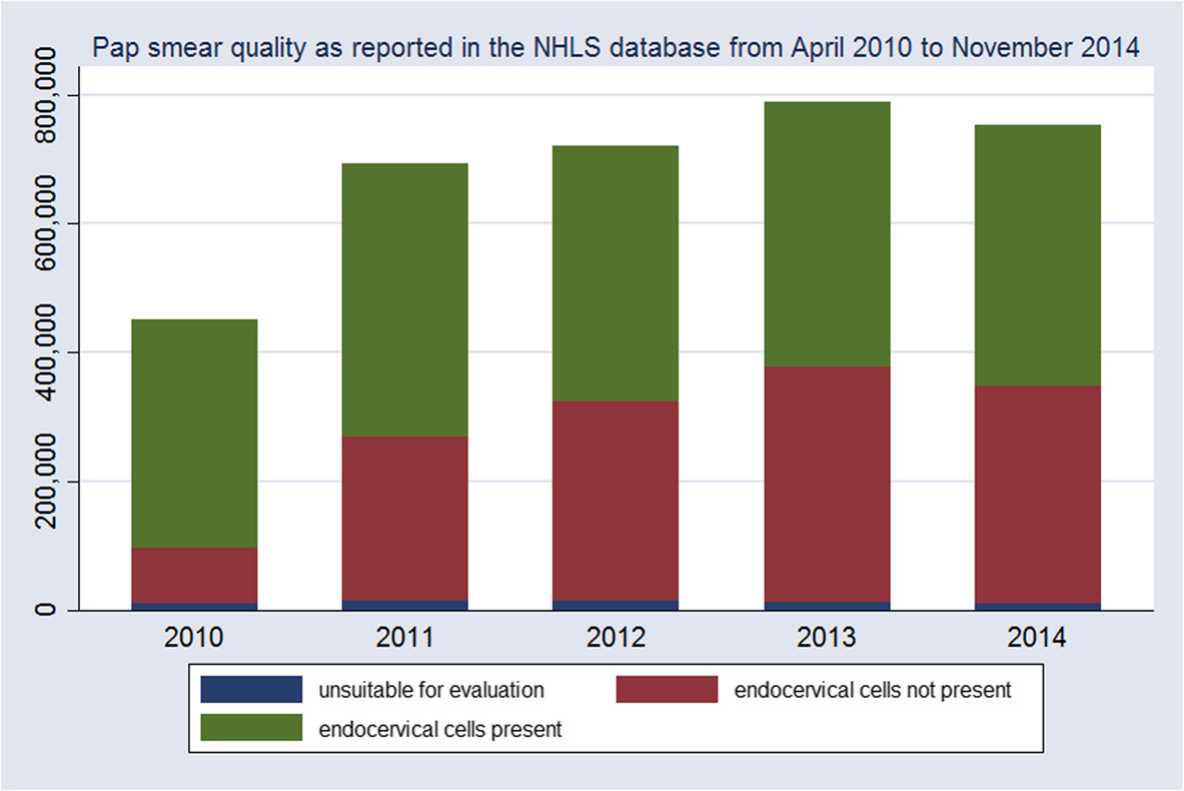
Counts of adequate and inadequate specimens evaluated by the NHLS, April 2010 to November 2014

Unfortunately, with the increase in testing volumes in the South African national cervical cancer screening programme, there has also been a decrease in the quality of Pap smears collected, as defined by the absence of endocervical cells. In 2010, 80.5 % of specimens submitted were evaluated as adequate for evaluation; in 2014, only 54.4 % of specimens met the same criteria. Figure 2 indicates the decreasing adequacy of specimens over time. There are multiple reasons for the poor adequacy rate of Pap smears submitted for conventional cytology in South Africa – overworked clinic and laboratory staff, lack of training, task shifting without training, and rapid increase in volumes of slides submitted. Literature on quality of Pap smears indicated that adoption of an improved collection device, such as the broom, could improve the collection of endocervical cells.

South African guidelines for Pap smears also indicate that the recommended device for specimen collection is the wooden spatula with an elongated tip (Aylesbury spatula). The NHLS provides the spatula, glass slides, slide fixative, and laboratory request form to the clinics. A Cochrane review focusing on cytological specimen collection devices suggests that the proportion of specimens that are satisfactory for evaluation and include endocervical cells may be improved if the spatula is replaced by either the cytobrush used in combination with the spatula (odds ratio (OR): 3.48, 95 % confidence interval (95 % CI): 3.20–3.78) or the Cervex-Brush (OR: 1.57, 95 % CI: 1.42–1.73], also known as the ‘broom’ [7]. The broom has parallel plastic fibres, with longer fibres in the middle and shorter fibres on the outside so that the endocervical and ectocervical cells can be collected simultaneously. The cytobrush (used to collect endocervical cells) and wooden spatula (used to collect ectocervical cells) combination involves more steps and consumables than use of the broom alone, thus the broom is the simpler option to implement. Currently, the elongated-tip wooden spatula is on public tender, and available for < $0.02 per spatula while the broom is available from private medical suppliers at $0.23 per broom. To investigate the potential cost-effectiveness as defined by the cost per high-grade lesion detected from a provider perspective of using the more expensive broom in the South African national cervical cancer screening programme for HIV-infected women, we created a decision-analysis model based both on the intended (guideline) and actual practice for collection and evaluation of Pap smears using national-level data from the NHLS and published research on true rates of positivity. Because of the larger volume of testing for HIV-infected women, and the higher risk of infection, persistence of infection and progression of dysplasia and cervical disease for HIV-infected women [3], the model focuses only on the HIV-infected population.

## Methods

### Definitions and guidelines

As indicated above, the South African cervical cancer guidelines define an adequate smear as one where both ecto- and endocervical cells are present. If any abnormality is seen, results are reported by the laboratory, even if it does not meet the minimum requirements for adequacy. According to South African cervical cancer screening guidelines, women should be recalled for immediate retesting if a negative Pap smear is found to lack endocervical cells or if it is unsatisfactory for evaluation because of other reasons, such as blood obscuring the sample [4]. This guideline differs from contexts where Pap smears are repeated more frequently, HPV testing is more widely used, or HIV-infection less prevalent. The following are considered abnormal Paps in the South African context: atypical squamous cells: unknown significance (ASC-US) or cannot exclude HSIL or high-grade changes (ASC-H); low-grade squamous intraepithelial lesion (LSIL); high-grade squamous intraepithelial lesion (HSIL); and squamous cell carcinoma. Women with atypical glandular cells of undetermined significance (AGC) are referred for specialist care. If HSIL or ASC-H [8] is reported, the woman should be referred for colposcopy or specialist care. Abnormal Pap smears which are ASC-US or LSIL should be repeated the following year and if diagnosis unchanged (e.g. persistent ASC-US) or worse, the woman should be referred for colposcopy. Human papillomavirus (HPV) testing is not routinely available within the South African public health sector.

### Model structure

A decision-analysis model was established to represent intended (guideline) practice for when an HIV-infected woman has a Pap smear done at a public health facility in South Africa. Screening guidelines were chosen as the base model for estimating the cost-effectiveness of the broom collection device as care that is does not meet guidelines can be considered ‘substandard’.

**Fig. 3:**
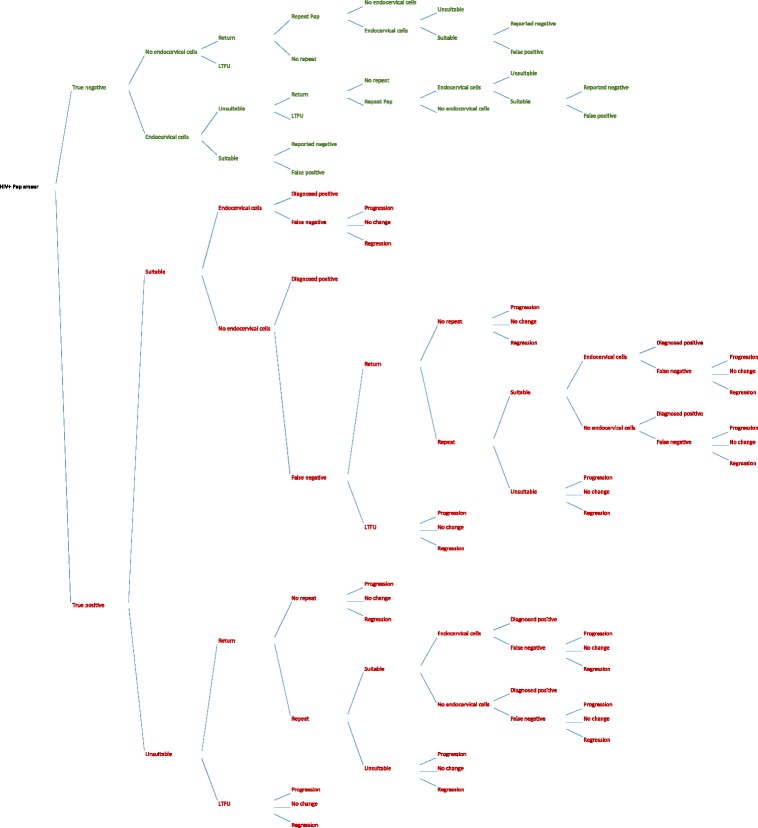
Decision analysis model, diagnosis of cervical dysplasia according to South Africa guidelines (intended practice)

Figure 3 shows the guidelines (intended practice) model. The start of the model is an HIV-infected woman receiving a Pap smear from a public health facility, she is either a true negative (normal Pap smear) or true positive (abnormal Pap smear). In the model, a woman who has no cervical dysplasia may be correctly diagnosed with a normal Pap smear, receive a false positive diagnosis (depending on the Pap test’s specificity), or be lost to care prior to receiving a diagnosis. Each of these different outcomes represents a different ‘branch’ of the decision-analysis model ‘tree’. A woman with cervical dysplasia may be correctly diagnosed with an abnormal Pap smear, receive a false negative diagnosis (depending on the Pap test’s sensitivity), or be lost to care prior to receiving a diagnosis. The cervical dysplasia in women who are not diagnosed (due to false negatives or loss to care) may regress, persist, or progress prior to her next Pap smear (3 years). The proportions of women with true disease, detected abnormal cells, and disease that progresses are differentiated according to grade: ASC-US, LSIL and HSIL (including ASC-H). AGC was not modelled as it represented 0.1 % of reports by the NHLS from 2010 to 2014.

As noted above, when the laboratory grades the specimen as inadequate, the woman should be rescreened. However, as can be seen in Fig. 3, some women may be lost-to-follow-up and never return to the clinic for a repeat Pap smear. Other women may return to the clinic, but the Pap smear is not repeated for reasons related to the clinic (e.g. inadequate staffing or supplies, no electricity, closing time) or reasons related to the patient (e.g. unwilling to repeat the examination, no time for repeat examination, menstruating). As above, the cervical dysplasia in women who are not diagnosed prior to being lost to care may regress, persist, or progress prior to her next scheduled Pap smear (3 years). In this model, a high proportion of inadequate slides leads to missed opportunities for diagnosis as women are lost to care. Inadequate slides also result in an increased burden on the patient, health care facility, and cytology laboratory from repeat testing.

**Fig. 4:**
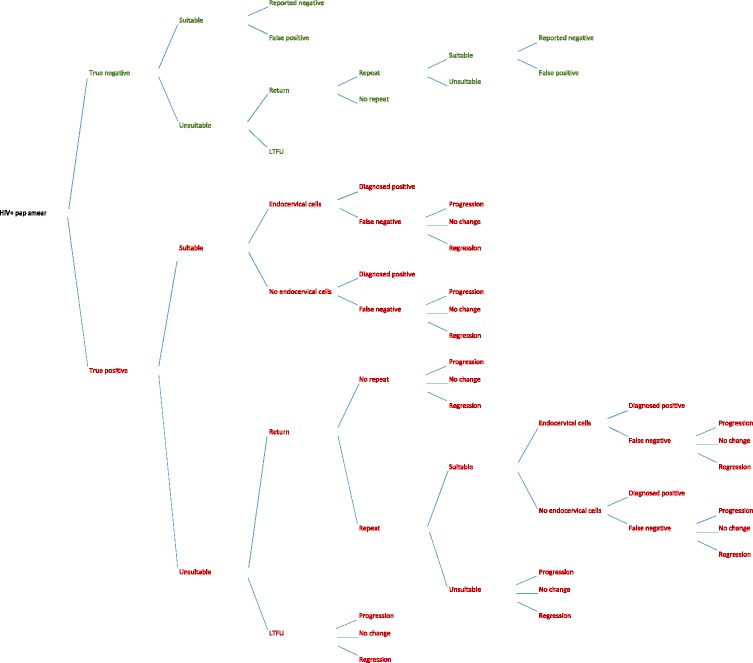
Decision analysis model, diagnosis of cervical dysplasia, typical practice in South Africa

Because of the difficulties in repeating the large number of Pap smears that are inadequate within the national programme, in many clinics typical practice differs from intended practice. Thus, an alternative scenario was constructed. We created a decision-analysis model that was structured to reflect typical practice (Fig. 4). Under typical practice, as under intended practice, all detected abnormalities are reported regardless of whether endocervical cells are present. However, if no abnormality is reported the Pap smear is not repeated, regardless of the lack of endocervical cells, even for women who may be at high risk of cervical disease. In this model, a high proportion of inadequate slides leads to a high number of false negative diagnoses as the sensitivity of Pap smears decreases when there are no endocervical cells present, as per results of the Cochrane review (OR: 1.89, CI 95 %: 1.79 – 2.00) [7]. As in the intended practice (guidelines) model, the cervical dysplasia in women who are not diagnosed due to false negatives may regress, persist, or progress prior to her next scheduled Pap smear (3 years).

Both models were built in Stata, v13 (College Station, TX, USA). The estimated probability of a woman at each outcome (branch) was multiplied by the cost of that outcome. Uncertainty around model parameters was evaluated using Monte Carlo simulation; 10,000 trials were run for each models. Mean values for key results were compared using linear regression. Neither costs nor outcomes are extended beyond screening for cervical cancer; the model is intended to provide information on the decision about the potential incremental cost and diagnoses though the use of two different collection devices for Pap smears; the cost-effectiveness of early detection of cervical dysplasia is assumed.

### Model parameters

Model parameters for the guidelines and typical practice models, including mean values, shape of sampled distribution, and 95 % confidence interval are reported in Tables 1 and 2.

**Table 1:** Model input parameters. Parameters used for intended and typical practice models

Parameter	Mean	CI 95 %		Distribution	Source
		p5	p95		
Women tested (annual count)	723,829	583,056	869,393	Normal	NHLS database
Presence of endocervical cells broom vs spatula, odds ratio	1.57	1.44	1.70	Normal	[7]
Probability of detection of atypia/dyskaryosis in smears with enodcervical cells vs those without, odds ratio	1.89	1.80	1.98	Normal	[7]
Probability of detection of severe atypia/dyskaryosis in smears with enodcervical cells vs those without, odds ratio	2.46	1.75	3.17	Normal	[7]
HIV-infected (%)	51	40	64	Binomial	NHLS database
HIV-infected women tested (annual count)	372,340	261,707	493,988	Normal	Calculated
Abnormal Pap smear (% of all smears)	73	71	75	Binomial	[9]
HSIL (% of all smears)	31	29	33	Binomial	[9]
LSIL (% of all smears)	37	35	39	Binomial	[9]
ASC-US (% of all smears)	5	4	6	Binomial	[9]
Endocervical cells present, spatula (% of all smears)	53	52	54	Binomial	NHLS database
Endocervical cells present, broom (% of all smears)	83	76	90	Binomial	Calculated
Sensitivity (%, if endocervical cells present)	76	72	80	Binomial	[9]
Sensitivity (%, if no endocervical cells present)	40	35	45	Binomial	Calculated
Sensitivity HSIL (%, if no endocervical cells present)	32	23	44	Binomial	Calculated
Repeat Pap smear done (% of patients)	90	85	95	Binomial	Assumption
Smear satisfactory for evaluation (% of all smears)	98	98	99	Binomial	NHLS database
Specificity (%)	84	81	87	Binomial	[9]
Lost to follow-up (% of patients)	8	6	10	Binomial	[10]

**Table 2:** Model input parameters. Cost parameters (2014 USD) used for intended and typical practice models

Parameter	Mean	Distribution	Source
USD/ZAR exchange rate 2014	$ 0.09	Point	[13]
Wooden spatula, elongated tip	$ 0.02	Point	NHLS 2014
Broom (cervex brush, type E)	$ 0.22	Point	Quotation 2014
Outpatient clinic visit (no bed facility)	$ 8.36	Point	[11], [12]
Laboratory test charges (excluding collection device)	$ 4.89	Point	NHLS 2014

Annual numbers of Pap smears evaluated, the proportion of specimens reported to have been collected from HIV-infected women, the number of slides with endocervical cells, and the number of slides not suitable for evaluation were extracted from the NHLS database of 3.6 million slides examined between April 2010 and November 2014.

True rates of abnormal Pap smears that are expected from an HIV-infected population at a public sector facility in South Africa were extracted from literature; sensitivity and specificity of Pap smears collected using broom with endocervical cells present were taken from the same study [9]. The proportion of women not returning to the clinic for a repeat Pap smear was based on the lost-to-follow-up rate from a South African study [10]. The rate of repeat Pap smears for those women who did return to the clinic was assumed.

Odds ratios of the presence of endocervical cells on a specimen collected by a broom compared to the elongated tip spatula and of the rates of detection of cervical dysplasia from smears with endocervical cells compared to those without were extracted from a Cochrane review of cervical collection devices for standard cytology [7].

Provider costs (costs incurred by the Department of Health to provide the Pap smear service) were estimated using WHO CHOICE ingredients-based approach [11]. NHLS provided the costs of the wooden spatula, which is purchased in large quantities on tender for < $0.02 each. A quotation was obtained for procurement of 100 broom devices for $0.23 each. In addition to the cytology laboratory test (NHLS charge of $4.89, having excluded the cost of the collection device), it was assumed that Pap smears are done within the context of an outpatient visit by the woman to a clinic. Therefore, the WHO CHOICE 2008 USD cost for an outpatient visit to no-bed public sector facility in South Africa [11] was inflated to 2014 values using annual consumer price index for the period [12]. The NHLS charge for a Pap smear and local quotation for the broom were converted to USD using the average 2014 exchange rate of ZAR 10.83/USD [13].

Costs for active follow-up of patients needing to be rescreened, e.g. phone calls or home visits, were excluded as practice and costs differ by clinic and geography of the clinic catchment area. Costs incurred by patients, e.g. paying for transport to the clinic, or opportunity costs for patients to visit the clinic multiple times, e.g. lost wages, were also excluded as the analysis is from a provider perspective only.

This study did not involve human subjects; no patient-level medical data or records were accessed or analysed. We report all results according to the Consolidated Health Economic Evaluation Reporting Standards (CHEERS) statement [14].

## Results

Each year, the NHLS receives an average of 723,829 gynaecological slides for conventional cytology; of these an average 372,340 per year will be from HIV-infected women.

For HIV-infected women, using the wooden spatula for specimen collection and implementing cervical cancer screening guidelines (intended practice) costs $6.25 million per year, or $16.79 per woman tested (Table 3). If the Cervex-Brush (broom) were used as the standard collection device, it could save the South African cervical cancer screening programme $0.67 million per year (95 % CI: $0.64 to $0.70 million), or $1.81 per woman tested (95 % CI: $1.79 to $1.82). This 11 % decrease in cost is obtained through increased collection of endocervical cells and consequent reduction in the need to repeat Pap smears. At the same time, use of the broom as the collection device for Pap smears from HIV-infected women could result in 7 % more abnormal slides reported, an absolute increase of 14,222 cases (95 % CI: 13,093 to 15,351), based upon the expected true Pap smear positivity from HIV-infected women. The majority of this increase would comprise of HSIL cases - 6,180 cases per year (8 %, 95 % CI: 5,276 to 6,634) - because of the increased sensitivity of Pap smears for HSIL when endocervical cells present. Most of the increase in detection of abnormalities results from an increase in diagnosis (30,837 more women, 95 % CI: 30,640 to 31,033) compared to high rates of lost to follow-up without repeating the Pap smear. Results are sensitive to assumed rates of women returning to the clinic when recalled and having a repeat Pap smear once at the clinic. Under intended practice, for each additional HSIL case detected among HIV-infected women, the South African cervical cancer screening programme could save $13.64 (95 % CI: $13.52 to $13.76) by using the broom as its standard of care collection device.

**Table 3:** Model results

	Spatula	Broom	Incremental difference		
			Value	%	95 % CI
Guidelines (intended practice)					
Annual programme cost	$ 6,246,919	$ 5,575,183	- $ 671,736	-11 %	-$ 702,963 to - $ 640,509
Cost per woman screened	$ 16.79	$ 14.98	- $ 1.81	-16 %	- 1.82 to -$ 1.79
Cost per HSIL case detected	$ 78.91	$ 65.27	-$ 13.64	-21 %	-$ 13.76 to -$ 13.52
Abnormal smears detected	204,181	218,403	14,222	7 %	13,093 to 15,351
HSIL smears detected	79,465	85,645	6,180	8 %	5,726 to 6,634
False negative smears reported	63,641	62,377	-1,264	-2 %	-1,631 to -897
No diagnosis (lost to care)	43,197	12,361	-30,837	-71 %	-31,033 to -30,640
Typical practice					
Annual programme cost	$ 5,034,091	$ 5,113,113	$ 79,022	2 %	$ 52,548 to $ 105,496
Cost per woman screened	$ 13.46	$ 13.67	$ 0.21	2 %	$ 0.20 to $ 0.22
Cost per HSIL case detected	$ 79.60	$ 65.05	-$ 14.55	-18 %	-$ 14.68 to - $ 14.42
Abnormal smears detected	172,421	204,604	32,184	19 %	31,178 to 33,189
HSIL smears detected	63,566	78,861	15,295	24 %	14,900 to 15,689
False negative smears reported	116,082	83,898	- 32,184	-28 %	-32,748 to 31,619
No diagnosis (lost to care)	1,223	1,223	0	0 %	-9 to 9

For HIV-infected women, according to the typical practice model, where Pap smears are rarely repeated even if no endocervical cells present on a normal slide, using the wooden spatula for specimen collection costs $5.0 million per year, or $13.46 per woman tested. If the broom were used as the standard collection device, this would cost an additional $79,022 per year (95 % CI: $52,548 to $105,496) or $0.21 per woman tested (+2 %, 95 % CI: $0.20 to $0.22) for the more expensive broom collection device. At the same time, use of the broom as the collection device for Pap smears could result in 19 % more abnormal slides reported, an absolute increase of 32,184 cases (95 % CI: 31,178 to 33,189) per year based upon the expected rates of Pap smear true positivity for HIV-infected women. The number of HSIL cases detected would increase by 15,295 per year (+24 %, 95 % CI: 14,900 to 15,689). Using the broom could also result in a 28 % decline (32,184 fewer women, 95 % CI: 31,619 to 32,748) in the number of women who receive a false negative diagnosis. Results are sensitive to current rates of adequacy in the national programme with the use of the spatula device and the gains in sensitivity of the Pap in detecting cervical dysplasia on slides with or without endocervical cells. Under typical practice, the screening programme could save $14.55 (95 % CI: $14.42 to $14.68) for each HSIL case detected if the broom was used.

## Discussion

South African HIV treatment guidelines indicate that HIV-infected women should have a Pap smear every three years from diagnosis of HIV [5]. With an estimated 3.4 million women living with HIV in South Africa, the number of Pap smears done each year in the public sector needs to almost triple from the current average of 372,340 per year. However, previous rapid expansion in Pap smear coverage corresponded with a decline in quality. The proportion of slides with endocervical cells submitted to the NHLS for cytology from public sector clinics declined, from 80.5 % in 2010 to only 54 % in 2014. The national cervical cancer screening guidelines set a target for public health facilities to have at least 70 % adequacy rate (slides with endocervical cells present). The concern is that, with the lower sensitivity of Pap smears when no endocervical cells are present, either a large number of Pap smears must be repeated (wasteful expenditure) or a large number of women receive false negative results and the benefits of having a Pap smear screening programme are not realized.

According to the decision analysis model and Monte Carlo simulation presented here, although the broom is more expensive than the wooden spatula currently used, for the HIV-infected women tested (45–55 % of all women) the South African cervical cancer screening programme could save $0.67 million each year by avoiding repeat Pap smears. In both the intended practice and typical practice models, for the detection of HSIL cases, the broom is both more effective and less expensive than the spatula.

The cost of a Pap smear was built from the cost of a clinic visit, the conventional cytology laboratory charge and the cost of the collection device. If a nurse spends more time with a woman to do a Pap smear than with a patient presenting at the outpatient facility for another reason, the costs here may be underestimated. This average cost may also underestimate the costs of serving those that are hard to reach: a Pap smear in rural communities in South Africa served by a mobile van costs $46 to $76 instead of the $16.79 estimated here [15]. As NHLS is a parastatal entity, laboratory charges are standardized across all public health facilities and are gazetted annually by the national government. Laboratory charges do not necessarily reflect the costs incurred by the NHLS to provide the service, but do reflect the cost to the Department of Health and the South African Treasury. Also, we did not include costs for follow-up of women to return to the clinic (e.g. calling the woman to return to the clinic, additional patients in the clinic, time to explain the need for a repeat visit). One study from the South African context estimates that this could be $2.27 to $7.62 per woman traced by the clinic [16]. Loss to follow-up may be higher than the 8 % used in the model (range 6–10 %) here, a study in rural South Africa found that 46 % of women referred for colposcopy did not attend their appointment [17]. We also only considered the costs from a provider perspective and did not include the costs women incur to (repeatedly) present to the clinic such as transport, lost wages, childcare, or food at the facility. Thus, both the provider cost and the societal cost of Pap smears are likely underestimated here. Further, the wooden spatula is currently on public tender for procurement of 1 million per year, while the cost used for the plastic broom was obtained from a quotation from a private medical supplier. It is likely that if the broom were procured for the public sector at the quantities currently required each year that the price could drop significantly. Therefore, the incremental cost savings for each woman screened under the intended practice model is conservative. For the typical practice model, the incremental cost per woman screened may be even less than $0.21 (2 %) currently estimated.

The model also presents a conservative view of the benefits of using the broom: increased detection of HSIL. Undiagnosed and untreated disease has the potential to progress and create an increasing burden for the health sector and society through the need to treat advanced disease and invasive cervical cancer. This is especially important for HIV-infected women with cervical dysplasia, as they are at a higher risk for persistent disease and faster disease progression [3]. This potential cost savings -- e.g. the savings from not having to treat cervical cancer with radiation, chemotherapy, specialist care and oncology, surgery and palliative care -- is not quantified here.

That said, the model is also limited to the costs and outcomes of the cervical cancer screening programme, it does not take into account the increased budget required for colposcopy or cancer treatment because more women were detected with cervical dysplasia rather than lost to care. However, as noted above, it is well established that identifying and providing early treatment for dysplasia is cost effective approach to managing cervical disease (i.e. as compared to managing late state cervical cancer).

One limitation of the study was that this was facility-level data and not patient-level; therefore, it was not possible to identify patient characteristics that may have led to increased risk of poor specimen collection. Thus, the recommendation has to be at programme level (adopt the broom for all Pap smears or all Pap smears for HIV-infected women) rather than for certain women. This needs more evaluation. Also, because the data was not patient level, it was not possible to identify whether the Pap smears recorded were initial or repeat Pap smears. In the models constructed, the probability that the repeat Pap would be adequate (satisfactory for evaluation and with endocervical cells present) was assumed to be independent from the initial Pap. It may be that women who had an initial inadequate Pap are more likely to have a second inadequate Pap, as the reasons for the first were not independent of the woman’s cervix or the clinic at which she receives care. However, it is also possible that a woman who had an initial inadequate Pap is more likely to have an adequate repeat Pap as the nurse taking the specimen is more careful on the repeat test or requests that a more experienced nurse take this repeat test. On average though, in the large national programme of cervical screening in South Africa, 46 % of Pap smears done in 2014 were inadequate and we believe that the models constructed can be used to guide policy as to reducing this number.

## Conclusions

Especially in resource-limited settings, policy makers are under pressure to procure lowest-cost items. Evidence as to the long-term consequences, including higher overall cost to the health system, of short-term savings is needed to change policies. Under a wide range of possible values for key parameters, the decision analysis model presented here for intended and typical practice indicate that use of a $0.22 plastic Cervex-Brush (broom) for collection of gynaecological specimens from HIV-infected women is a cost-effective choice compared to the cheaper wooden spatula (<$0.02 per device). The improved sensitivity of conventional Pap smears from better specimen collection by the broom, e.g. more slides with endocervical cells, could save the South African cervical cancer screening programme $13.64 to $14.55 for each case of HSIL detected among HIV-infected women.

## Abbreviations

AGC: Atypical glandular cells of undetermined significance
ASC-H: Atypical squamous cells cannot exclude HSIL or high-grade changes
ASC-US: Atypical squamous cells unknown significance
CI: Confidence interval
HIV: Human immunodeficiency virus
HPV: Human papillomavirus
HSIL: High-grade squamous intraepithelial lesion
LSIL: Low-grade squamous intraepithelial lesion
NHLS: National Health Laboratory Service
WHO: World Health Organization

## References

[1] South Africa [http://globocan.iarc.fr/Pages/fact_sheets_population.aspx?country=710##]. Accessed 12 January 2015.

[2] HIV estimates with uncertainty bounds: 1990-2013 [http://www.unaids.org/en/resources/documents/2014/HIV_estimates_with_uncertainty_bounds_1990-2013]. Accessed 12 January 2015.

[3] Denslow S, Rositch AF, Firnhaber CS, Ting J, Smith JS (2014). Incidence and progression of cervical lesions in women with HIV: a systematic global review. Int J STD AIDS.

[4] South African National Department of Health: National Guideline for Cervical Cancer Screening Programme. Pretoria: South African National Department of Health; 2000.

[5] South African Department of Health: Clinical Guidelines for the Management of HIV and AIDS in Adults and Adolescents. Pretoria: South African National Department of Health; 2010.

[6] Solomon D, Nayar R. (Eds): The Bethesda System for Reporting Cervical Cytology: Definitions, Criteria, and Explanatory Notes. 2nd edition. Springer ISBN-13: 978-0387403588; 2001.

[7] Martin-Hirsch P, Jarvis G, Kitchener H, Lilford R. Collection devices for obtaining cervical cytology samples. Cochrane Database Syst Rev 2000(3):Art. No. doi:10.1002/14651858 CD001036. 10.1002/14651858.CD001036 10908482

[8] Michelow PM, Hartman I, Schulze D, Lamla-hillie S, Williams S, Levin S, Firnhaber CS (2010). Atypical squamous cells, cannot exclude high grade squamous intraepithelial (ASC-H) in HIV-positive women. Cytopathology.

[9] Firnhaber CS, Mayisela N, Mao L, Williams S, Swarts A, Faesen M, Levin S, Michelow PM, Omar T, Hudgens MG, Williamson A, Allan B, Lewis DA, Smith JS (2013). Validation of cervical cancer screening methods in HIV positive women from Johannesburg South Africa. PLoS One.

[10] Denny LE, Boa R, Williamson A-L, Allan BR, Hardie D, Stan R, et al. Human papillomavirus infection and cervical disease in Human Immunodeficiency Virus-1 – infected women. Obstet Gynecol. 2008;111:1380–7. 10.1097/AOG.0b013e3181743327 18515522

[11] Choosing Interventions that are Cost-Effective. WHO-CHOICE unit cost estimates for service delivery - Estimation file. 2011.

[12] Bureau of Labor Statistics: Consumer Price Index. 2014.

[13] Historical Exchange Rates [http://www.oanda.com/currency/historical-rates/]. Accessed 12 January 2015.

[14] Husereau D, Drummond M, Petrou S, Carswell C, Moher D, Greenberg D, Augustovski F, Briggs AH (2013). Consolidated Health Economic Evaluation Reporting Standards (CHEERS) Statement. Value Heal.

[15] Schnippel K, Lince-Deroche N, van den Handel T, Molefi S, Bruce S, Firnhaber C. Cost evaluation of reproductive and primary health care mobile service delivery for women in two rural districts in South Africa. PLoS One. 2015;10:e0119236. 10.1371/journal.pone.0119236 PMC4353629 25751528

[16] Goldhaber-Fiebert JD, Denny LE, De Souza M, Kuhn L, Goldie SJ (2009). Program spending to increase adherence: South African cervical cancer screening. PLoS One.

[17] Knegt Y (2014). Audit of cervical cancer screening and colposcopy attendance in rural South Africa. Afr J Reprod Health.

